# Improving the Usability of Written Exposure Therapy for Therapists in the Department of Veterans Affairs Telemental Health: Formative Study Using Qualitative and User-Centered Design Methods

**DOI:** 10.2196/47189

**Published:** 2023-11-06

**Authors:** Megan Moldestad, Valentina V Petrova, Katie Tirtanadi, Sonali R Mishra, Suparna Rajan, George Sayre, John C Fortney, Heather Schacht Reisinger

**Affiliations:** 1 Seattle-Denver Center of Innovation for Veteran-Centered and Value-Driven Care VA Puget Sound Health Care System Seattle, WA United States; 2 Department of Human-Centered Design and Engineering University of Washington Seattle, WA United States; 3 Department of Internal Medicine Division of Cardiology University of Michigan Ann Arbor, MI United States; 4 School of Public Health University of Washington Seattle, WA United States; 5 Department of Psychiatry and Behavioral Science School of Medicine University of Washington Seattle, WA United States; 6 Center for Access and Delivery Research and Evaluation Iowa City VA Health Care System Iowa City, IA United States; 7 Department of Internal Medicine University of Iowa Iowa City, IA United States

**Keywords:** evidence-based psychosocial interventions, telehealth, qualitative, user-centered design, implementation science, Department of Veterans Affairs health care system

## Abstract

**Background:**

User modifications are common in evidence-based psychosocial interventions (EBPIs) for mental health disorders. Often, EBPIs fit poorly into clinical workflows, require extensive resources, or pose considerable burden to patients and therapists. Implementation science is increasingly researching ways to improve the usability of EBPIs before implementation. A user-centered design can be used to support implementation methods to prioritize user needs and solutions to improve EBPI usability.

**Objective:**

Trauma-focused EBPIs are a first-line treatment for patients with posttraumatic stress disorder (PTSD) in the Department of Veterans Affairs. Written exposure therapy (WET) is a brief, trauma-focused EBPI wherein patients handwrite about trauma associated with their PTSD. Initially developed for in-person delivery, WET is increasingly being delivered remotely, and outcomes appear to be equivalent to in-person delivery. However, there are logistical issues in delivering WET via video. In this evaluation, we explored usability issues related to WET telehealth delivery via videoconferencing software and designed a solution for therapist-facing challenges to systematize WET telehealth delivery.

**Methods:**

The *Discover, Design and Build, and Test* framework guided this formative evaluation and served to inform a larger Virtual Care Quality Enhancement Research Initiative. We used qualitative descriptive methods in the *Discover* phase to understand the experiences and needs of 2 groups of users providing care within the Department of Veterans Affairs: in-person therapists delivering WET via video because of the COVID-19 pandemic and telehealth therapists who regularly deliver PTSD therapies. We then used user-centered design methods in the *Design and Build* phase to brainstorm, develop, and iteratively refine potential workflows to address identified usability issues. All procedures were conducted remotely.

**Results:**

In the *Discover* phase, both groups had challenges delivering WET and other PTSD therapies via telehealth because of technology issues with videoconferencing software, environmental distractions, and workflow disruptions. Narrative transfer (ie, patients sending handwritten trauma accounts to therapists) was the first target for design solution development as it was deemed most critical to WET delivery. In the *Design and Build* phase, we identified design constraints and brainstormed solution ideas. This led to the development of 3 solution workflows that were presented to a subgroup of therapist users through cognitive walkthroughs. Meetings with this subgroup allowed workflow refinement to improve narrative transfers. Finally, to facilitate using these workflows, we developed PDF manuals that are being refined in subsequent phases of the implementation project (not mentioned in this paper).

**Conclusions:**

The *Discover, Design and Build, and Test* framework can be a useful tool for understanding user needs in complex EBPI interventions and designing solutions to user-identified usability issues. Building on this work, an iterative evaluation of the 3 solution workflows and accompanying manuals with therapists and patients is underway as part of a nationwide WET implementation in telehealth settings.

## Introduction

### Background

Evidence-based psychosocial interventions (EBPIs) for mental health disorders often have poor uptake in clinical settings as they do not fit well into existing workflows, require extensive facility resources, or pose a considerable cognitive burden on users (ie, patients or therapists) [1]. User modifications are common in attempts to align EBPIs more closely with local contexts and clinical workflows [2]. Although much of implementation science has taken a more top-down approach focused on how to adapt clinical contexts for EBPI uptake, a burgeoning focus is to work with users in a more bottom-up approach to adapt EBPIs to improve their usability in local contexts [3-5]. Usability is the degree to which a program or intervention can be used by certain users easily, efficiently, and with low user burden and high satisfaction [1].

Given the variability in local contexts, systematically increasing fit requires approaches to understanding workflows and contexts that are simultaneously rigorous, responsive, flexible, and solution focused. User-centered design (UCD) is one such approach, and although UCD methods have their origins in product development, they are increasingly being explored by implementation scientists to improve EBPI usability regardless of whether the EBPI involves a product in the traditional technological sense (eg, user interface or app) or a process (eg, clinical workflow) [2,5]. Often incorporating qualitative data collection and analysis methods, UCD aims to understand the needs, preferences, and experiences of users; the processes through which they complete certain tasks; and the contexts in which they work, all with an eye toward producing actionable results [4].

The Department of Veterans Affairs (VA) is the largest integrated health care network in the United States [6], and 11% of VA users have posttraumatic stress disorder (PTSD; personal communication with Harpaz-Rotem and R Hoff). Trauma-focused EBPIs are considered the first-line treatment for PTSD [7], and although the VA widely delivers prolonged exposure (PE) and cognitive processing therapy (CPT), these treatments are generally offered in specialty PTSD clinics and have historically low completion rates [8]. Multiple studies highlight the increasing awareness of problems with the design of EBPIs that limit their use in clinical practice, including unsuccessful referrals or handoffs, low ease of use, and high dropout because of poor fit with the intended context [2].

Written exposure therapy (WET) [9-11] is a relatively new, brief, trauma-focused EBPI recommended as a first-line treatment for PTSD [7]. Initially developed for in-person delivery, it involves patients handwriting about a traumatic experience associated with their PTSD while in session with a therapist. After an introductory session orienting the patient to the therapy, each therapeutic session begins with the therapist reviewing the patient’s handwritten narrative from the previous session and coaching them through what to focus on in the current session to work through the identified trauma. Similar to other trauma-focused EBPIs such as PE and CPT, WET retains exposure to trauma as a core element. However, WET does not require patient homework (thus eliminating 1 element of avoidant behavior typical in patients with PTSD); is briefer (fewer and shorter sessions than PE or CPT); and requires considerably less therapist time, training, and supervision to administer [12]. Thus, WET has the potential for widespread adoption in the VA.

In a quantitative study of WET’s effectiveness in routine VA care settings (both in-person and video-based telehealth), the results showed that patients had significant improvements in PTSD and depression symptoms and decreases in functional impairment [8]. Worley et al [12] found that, although the COVID-19 pandemic did increase the dropout rate of WET, it was marginal (from approximately 20% to 27%). In a mixed methods study conducted during the pandemic and focusing on patient data [13], the findings revealed that attrition rates were slightly higher (33%) in their sample of nonveteran, predominantly Latino patients, yet patients endorsed telehealth via videoconferencing as similar to in-person service delivery. In all studies, delivering WET via videoconference-based telehealth introduced unique clinical workflow problems, such as therapists obtaining handwritten trauma accounts before the next session, or barriers to delivery, such as distracting home environments or poor internet service. Understanding the nuances of these challenges and developing solutions is necessary to bolster the usability and effectiveness of WET delivered via videoconference-based telehealth.

### Objectives

Our objective was to (1) add to the knowledge base of WET delivered via videoconference-based telehealth and (2) incorporate aspects of a UCD framework to explore and improve WET usability for one group of users (ie, therapists) in this context. The formative work presented in this paper is part of a large ongoing evaluation known as the Virtual Care Quality Enhancement Research Initiative (QUERI) program. The Virtual Care QUERI implements and evaluates EBPIs that incorporate non–in-person care technologies to improve access to high-quality care for rural veterans. Thus, WET delivered via videoconference-based telehealth is one of several EBPIs being evaluated as part of this QUERI. Importantly, although the Virtual Care QUERI subproject from which this study comes is focused on promoting WET telehealth adoption by both therapists and patients, the work presented in this paper focuses only on therapists. This was done because, in the initial stages of the project, we first sought to understand the limits of VA therapist workflows and care delivery systems and have therapists help identify characteristics of potential patient users for the subsequent patient-facing phase of the project. To our knowledge, this is the first qualitative study of therapist experiences with WET in the VA and the first to use a UCD approach to improve the usability of its delivery.

## Methods

### Methodological Framework

The formative work described in this paper was guided by the *Discover, Design and Build, and Test* (DDBT) framework [1]. As the evaluation project is ongoing, only the work guided by the first 2 phases of the DDBT is described. DDBT draws from human-centered and UCD methods of understanding user needs and developing solutions in support of modifying and improving EBPIs. How we used the *Discover* and *Design and Build* phases of DDBT in relation to our work is described in the subsequent sections. The study by Lyon et al [1] provides a full description and a visual representation of the DDBT framework.

### Ethical Considerations

Per the Veterans Health Administration Program Guide 1200.21 [14], all authors attest that the activities that resulted in producing this manuscript were not conducted as part of a research project and, thus, did not require human participant research ethics review. The activities were conducted as part of a nonresearch, quality improvement evaluation (Virtual Care QUERI—QUE 20-007) conducted under the authority of the VA Office of Rural Health. Nonresearch designation was granted on April 26, 2019. All participants volunteered their time and verbally consented to take part in the evaluation. Per Veterans Health Administration regulations, participants were not compensated for their time. Identifying information has been removed to protect participant anonymity.

### Project Team

Senior author HSR, a medical anthropologist and health services researcher with relevant expertise in qualitative and formative evaluation methods, oversaw study procedures. Author KT, a health systems engineer with relevant expertise in UCD, provided extensive consultation on UCD methodology. Our interdisciplinary team had a range of experience with qualitative methods and analysis, implementation science, UCD, and VA health services research.

### Discover Phase: Understanding Therapist Users and Context

#### Overview

According to the DDBT framework, the *Discover* phase is the essential first phase in any project that aims to identify the needs and preferences of users, understand the context in which the EBPI is being delivered before formal adaptation, and clarify issues and barriers affecting usability [1]. Put simply, in the *Discover* phase, users are identified, their context is understood, and their needs are documented. Although users are most frequently therapists and patients [1], as mentioned in our objective, given the focus on therapist workflows and care delivery system barriers, therapists are the focus of this study. Thus, only the *Discover* phase methods and findings related to this group of users are outlined in the following sections.

We used a qualitative descriptive approach to sampling VA mental health therapists, data collection, and analysis [15,16]. Qualitative descriptive methods are rooted in naturalistic inquiry [17], which is a useful theoretical framework when a description of the phenomena under study is the primary goal of inquiry [18]. As we wanted to identify usability issues as described by our groups of therapist users, having a practical theoretical approach guiding our work was essential. Our sampling plan was designed to target therapists with experience with (1) WET or (2) delivering other PTSD therapies via videoconference-based telehealth. We wanted to focus our understanding on these groups’ workflows; the barriers to and facilitators of clinical care delivery in the aftermath of the COVID-19 pandemic; and the issues most affecting (or likely to affect) WET usability from the therapists’ perspective, including adaptations to delivering WET via video. COVID-19 pandemic constraints and VA travel restrictions prevented us from observing users in person, so in lieu of structured observations [1], during the interviews, we asked therapists to describe and show us their workspaces, including technologies used, to better understand their delivery context. We collected all data via video (using Teams [Microsoft Corp] or a digital audio recorder). Interviews with therapists were audio recorded and, along with descriptive observations of their workspaces, professionally transcribed.

#### Data Sources and Methods

##### User Group 1: WET Therapists

We interviewed a national sample of 10 PTSD therapists who were already trained in WET and actively providing the therapy to their patients. They will be referred to as the *WET* group. The WET group was not originally part of our intended sample but became so given COVID-19 pandemic in-person encounter restrictions at the time of data collection (February 2021 to April 2021). Specifically, we were interested in how this group, who had received training for in-person WET delivery, had provided WET via videoconference-based telehealth during the pandemic. Interview guides and follow-up probes (Multimedia Appendix 1) focused on WET delivery in a telehealth context, processes for routing patients to therapy, and understanding therapists’ ad hoc home workspaces via descriptions and demonstrations using their webcams.

##### User Group 2: Clinical Resource Hub Therapists

We interviewed a national sample of 14 telehealth therapists who were not trained in delivering WET but some of whom delivered other evidence-based PTSD therapies (eg, CPT and PE) via video to veterans. These therapists were associated with Clinical Resource Hubs (CRHs) [19] and are referred to as the *CRH* group. CRHs, or simply *Hubs*, connect underserved VA medical facilities through a hub and spoke model. Hubs are typically large VA medical centers, and spokes are smaller, community-based outpatient clinics. Veterans can receive care from hub therapists via videoconferencing telehealth technology either at spoke sites or from their homes. CRH therapists exclusively provide telehealth care via the VA’s designated clinical teleconferencing software, known as VA Video Connect. Owing to pandemic restrictions at the time of data collection (May 2021 to July 2021), many CRH therapists were working from home at the time that we interviewed them or, in some cases, split their time between working from home and working from a VA hub.

Although this group did not deliver WET therapy at the time of the interviews, we were interested in understanding their daily workflows and clinical processes given that they were already delivering other EBPIs in a completely web-based environment (via videoconferencing software) before the pandemic. We used their experiences to understand telehealth issues more broadly and, as is common in UCD in preparation of brainstorming solutions [20], to understand *how they might* do WET via video to get a sense of potential usability issues in the implementation of WET delivered via videoconferencing software. The interview guides and follow-up probes (Multimedia Appendix 2) focused on CRH therapists’ web-based workflows and setup.

At the end of each interview, we asked these participants if they were interested in being trained in WET delivery, and those who expressed interest were signed up for the next WET training cohort and worked with us in our subsequent project phase (*Design and Build*).

##### Other Data Sources

In addition to interviews and informal observations with our primary users (ie, WET and CRH therapists), our health systems engineer and UCD expert, KT, observed prerecorded WET training videos and spoke with secondary stakeholders (ie, WET developers) to further understand the processes and context for WET. No formal data (ie, recorded or transcribed) were collected from these observations or conversations, but instead, they served to provide contextual information for interviews and informal observations with primary users.

#### Analysis

We performed deductive and inductive content analysis [21] on our *Discover* phase data. Transcripts were uploaded to ATLAS.ti for Windows (version 9; ATLAS.ti Scientific Software Development GmbH) [22] for data management. Authors MM, VVP, CS, SR, and HSR (all with varying levels of qualitative method experience) met weekly from August 2021 to January 2022 to complete coding and analysis. We began coding on 1 WET and 1 CRH transcript as a group (ie, synchronously during meetings), starting with a list of deductive codes based on our project aims and inductive codes based on interview notes with primary users and conversations with secondary stakeholders. Code names and definitions were discussed as a group and updated according to our data, and new inductive codes were added to text that was not represented by previously developed codes. We coded 5 transcripts (2 WET and 3 CRH) in this way, which allowed us to (1) develop a codebook consisting of 20 total codes and (2) ensure consistency among coders. The remaining transcripts were split among coders, and coders were encouraged to create analytic memos during the coding process to capture personal reflections on emerging analyses and connections across the data set. After coding was complete, the team met regularly to discuss memos and codes and categorize the findings. We considered the analysis complete when we identified distinct categories that captured the data relevant to improving the usability of WET from the therapists’ perspective and continued coding until each category reached saturation [21,23].

## Results

### Overview

Our findings largely focus on the therapists’ perceptions of the suitability and determinants (ie, barriers and facilitators) of delivering WET via videoconferencing software. Experiences of actual WET delivery are expressed by WET therapists, whereas experiences of similar workflows or adjacent processes indicative of hypothetical WET delivery are expressed by CRH therapists. We organized these findings into four categories: (1) the general suitability of WET, (2) the suitability of WET delivered via videoconference-based telehealth, (3) common therapy session disruptions in a web-based environment, and (4) challenges and adaptations to existing workflows for WET delivered via video.

### General Suitability of WET

Before addressing the perceived suitability of WET delivered via videoconference-based telehealth, it is important to understand the perceived suitability of WET for veteran patients. WET therapists found the therapy to be an especially good fit for patients who are willing to proactively address their trauma. This perceived suitability was augmented by the therapy’s briefer nature compared with CPT or PE and the lack of between-session work (ie, homework), which WET therapists considered “less intimidating” than other common PTSD therapies:

I think the people who do best in this treatment are really good at addressing their avoidance on their own. Because once you get them writing, they’re off. And so, I think people who do the best, especially because the treatment is so short, are the ones who can say “I am going to address this memory, or I am going to approach my feelings.”

P3; WET

I think one reason people opt in [to WET] is that it seems less intimidating. It’s less time than the other therapies. They know that they’re going to get in there and it’s only five sessions...they’re going to get it done in [the] session, they’re not going to be dragging it out outside of session.

P4; WET

One therapist stated that some clients found writing rather than talking about trauma to be more in line with their cultural beliefs about sharing emotional information:

I’ve had a couple of my...Latino clients...what they’ve explained to me is it’s just kind of cultural beliefs about how and when emotions are shared, they didn’t feel okay emoting in therapy, but they felt like writing had a degree of privacy that sort of made sense for them, and again, their cultural rules about what you show or don’t show. So that was kind of a surprising benefit of [WET].

P2; WET

Not surprisingly, WET was not regarded as a good fit for all patients. For example, the therapists we spoke with expressed that those who avoid writing about their index trauma (ie, the traumatic focal point of the therapy), those who may have wrist or hand injuries, or those who prefer writing for enjoyment and do not want the therapy to “take writing away” may be less suited for WET:

I can think of one person in particular who declined because she was like, “I really enjoy writing and I have a feeling I’m going to hate therapy, and I don’t want it to take writing away from me.”...Another was an OEF/OIF (Operation Enduring Freedom/Operation Iraqi Freedom) veteran who had an injury to his wrist, so he ended up opting for PE, but then didn’t really do it.

P9; WET

### Suitability of WET Delivered via Videoconference-Based Telehealth

A few WET therapists expressed that this therapy was well suited for videoconference-based telehealth. A subset of WET therapists expressed a preference for WET video telehealth delivery, especially considering in-person logistical challenges because of COVID-19 mitigation measures during the pandemic. For example, 1 therapist talked about offering both in-person and telehealth WET in the future but stated having a personal preference for the latter given the difficulty in reading facial expressions on patients wearing surgical masks to prevent the spread of COVID-19. Another expressed an advantage to telehealth as patients can write in the presumed comfort of their own homes but with video and the therapist can still monitor what patients are doing:

Quite frankly, I’m happy to do VVC (VA Video Connect) forever...I get it, in some ways, it feels a little bit impersonal, but the alternative is sitting in an office doing therapy with someone with a mask on. The number of times I’ve been like, “I think you’re smirking, but I can’t tell because I can’t see your face, what is happening?” That’s super frustrating.

P9; WET

However, not all WET therapists preferred WET delivered via videoconference-based telehealth. Some expressed that they would “so rather do it in person” given the difficulty in obtaining narrative transfers from patients, which is explored in greater detail in the following sections.

CRH therapists reported that patients who are actively suicidal, have substance use issues, do not show up for appointments, push boundaries, do not want to do telehealth, or have internet connectivity issues would likely not be good candidates for WET delivered via video:

So, what would be a referral that wouldn’t be appropriate for your team?

Interviewer

Probably just flagrant substance use disorder, where it’s obvious they’re not hitting appointments. A lot of no-shows, a lot of cancellations. Yeah, it’s pretty obvious that their primary issue, we could do concurrent treatment for concurrent diagnosis, but not if they’re not showing up for it.

P17; CRH

Someone who’s actively suicidal [wouldn’t be appropriate]. [Or] someone who’s not willing to meet over telehealth.

P16; CRH

### Common Therapy Session Disruptions in a Web-Based Environment

Although not unique to WET, both WET and CRH therapists reported that common disruptions could affect WET delivery in a telehealth context. Both groups reported that, when disruptions do happen, they usually are on the patient’s end. The first type of disruption described was related to the patient’s physical privacy (eg, when someone walked into the patient’s therapy space during a session). Both therapist groups talked about setting expectations with patients up front and addressing expectations again as needed, including that they should have a private, quiet space from which to complete sessions:

I mean with distraction, there’s distractions all the time. Sometimes peoples’ phones ring, or somebody walks through the room. And so, I mean we all try to say, we always prompt them, “you need a private place, turn off any things.” And you do the best you can.

P3; WET

[One patient] said he was alone, he had privacy, and he’s in the middle of writing and then his wife walks through the room, and his grandson walks into the camera...obviously no privacy.

P7; WET

The second type of disruption that WET and CRH therapists shared was related to avoidance. Although also not unique to WET, therapists found that patients would avoid therapy in various ways. These included not setting up needed technology; not showing up for their appointments; or engaging in distracting activities during scheduled therapy time, such as driving or walking through a store:

Verbally [this patient] was paying a lot of lip service to being very committed to doing the treatment, but his actions suggested otherwise. And my interpretation was [he had] really active avoidance and maybe some poor insight into that. He didn’t have My HealtheVet (i.e., VA’s patient portal system, used to securely transmit the written narrative to the therapist). I gave him the instructions, I kept checking to see if he had signed up in the MyHealtheVet system. I wouldn’t see his name popping up. Eventually he did sign up for it, so that was good, it seemed promising. He sent me his writing then, [but] then after that it was canceling or no-showing appointments.

P7; WET

As with privacy-related disruptions, both groups of therapists talked about being direct with patients to address issues and attempt to remedy the distractions:

Sometimes, like all of a sudden, they’re driving or something, [and] I have to terminate. And they know that. I tell them, “If you’re ever driving, it’s me hanging up, because that’s a safety issue.”...But if [it feels preplanned], it’s felt more challenging, [so] just being more direct [with] them, and just saying “hey, this feels like avoidance, or it feels like you’re kind of distracted and not giving the session your whole attention.”

P14; CRH

The third and final type of disruption to therapy sessions was related to technology challenges (eg, internet connectivity and audio or video problems). For both WET and CRH therapists, internet connectivity was perhaps the biggest challenge, especially on the patients’ end or when therapists were working from home and using a web-based private network to connect to VA technologies and infrastructure. Although workarounds were possible—such as using phones for the session—these were seen as “not ideal”:

One thing, too, that I’ve run into with the people that we serve is [an unreliable Internet] connection, that’s probably the biggest interruption and the biggest challenge. Sometimes half of the session is spent just trying to get a good connection.

P16; CRH

I don’t know if this is accurate, my thought is that most of us are connecting (remotely) through VA VPN stuff. I feel like going from my house through the VA, to a veteran, the VA bandwidth, and the VA technology, that sort of leap is one of the major sources of lags and delays.

P10; WET

### Challenges and Adaptations to Existing Workflows for WET Delivered via Video

In addition to the aforementioned challenges, the primary barrier to smooth non–in-person care delivery involved transferring documents between patients and therapists. Specific to WET, an essential aspect of the protocol involves having patients handwrite about their index trauma during a session, which therapists then review before the subsequent session to provide feedback and guidance for future written accounts. In a face-to-face session, patients hand their narrative to therapists at the end of the session, which is securely stored until the subsequent session and then destroyed. In a telehealth session, this is not as straightforward, and WET therapists found this to be the greatest barrier to successful implementation. As the written narrative contains highly sensitive information, transmission via relatively insecure methods such as mail, email, or SMS text message creates the potential for negative consequences if there is a loss of confidentiality and data security:

The biggest thing is just how they’re going to get those dang accounts to me. And I would say that’s been the biggest hiccup with (virtual) Written Exposure Therapy, making sure I get the [handwritten narrative] account in between sessions.

P2; WET

I wish that there was some way that VVC (VA Video Connect) could capture their narratives right then and there, so that I just had them. All the worry would be gone of trying to get this narrative.

P3; WET

Although a small number of WET therapists described making rare exceptions to handwriting (eg, allowing patients to type or read aloud their narratives) to bypass challenges with narrative transfers, most did not make such deviations from this core element of the protocol. For CRH therapists, the process of obtaining requisite assessment paperwork (eg, measurement-based care instruments used to assess symptom severity) from patients was akin to obtaining handwritten narratives from patients during WET. Both CRH and WET therapists described using various tools for sharing documents with varying degrees of success. For measurement-based care instruments, the option that CRH therapists reported was most successful was when patients were seen in a VA facility (community-based outpatient clinic or inpatient setting) where a telehealth coordinating technician could give the patient a paper questionnaire and send the therapist a scanned copy of the filled-out version via encrypted email:

...if they’re in a clinic, it’s great, the TCT gives it to them, they fill it out, they email it back to me.

P22; CRH

Sometimes...I will send the documents as an attachment to the telehealth technician, and they’ll send it back to me. The veteran will [complete] it there in the clinic and [the TCT] will send it back to me in an encrypted email.

P24; CRH

The most secure—yet challenging—option for sending patient measures or other materials and getting things back from them was via My HealtheVet, the VA’s patient portal system. Attachments to secure messaging can only be accessed by the therapist team and are not part of the medical record. If patients were already set up with a My HealtheVet premium account, this was less challenging, and 1 WET therapist talked about how having this setup was now a local requirement for starting WET delivered via videoconferencing software:

If [patients] don’t have secure messaging set up it’s a nightmare...so actually, that’s kind of what we require now, is that [patients] have secure messaging set up before they start Written Exposure Therapy.

P3; WET

I can also send [documents] through My HealtheVet, but usually...people struggle with that.

P19; CRH

Some CRH therapists revealed that they did not use secure messaging for a variety of reasons. For example, 1 CRH therapist explained that they were concerned that it could be overwhelming to log in and track the volume of messages across the many sites they covered, and another mentioned that it could be complicated to set up. However, most of the reported challenges stemmed from what participants described as the patient-facing side of using secure messaging. For example, as many patients reportedly struggle with technology, without assistance at home, secure messaging either is not used or is only used with a fair amount of delay in getting documents back to therapists. However, obtaining help from family members or friends poses privacy concerns.

For patients who could not use My HealtheVet, both WET and CRH therapists had a variety of tools and workarounds to send and receive sensitive information. WET therapists described asking patients to hold narratives up to their cameras so that therapists could read on-screen in real time or take screenshots to read later. Although a seemingly simple solution, this was often fraught with problems owing to poor video quality:

The hardest one was the veteran who would hold it up to the screen. That was really challenging. Because sometimes it’s absolutely fine, and sometimes the video quality is terrible.

P1; WET

The least secure—but also the easiest—option was having patients send materials back via email. WET and CRH therapists reported obtaining permission from local leadership to use this option but only as a temporary solution until patients could get set up with My HealtheVet or a similarly secure, VA-approved platform:

[The patients send the narrative] through typical email. They take a photo and send it...Because attaching the email through secure messaging, attaching the picture to secure messaging is difficult for them to do.

P5; WET

I had one [patient] who did opt for that, but it was through email, and I tried really hard to convince her to just do the screenshot, and I think she got frustrated because it was so hard to get the camera to focus right...She just decided [she was] fine with emailing it. We got [local leadership] permission for email.

P6; WET

### Discover Phase: Summary

We used these interview findings as well as training observations and conversations with WET developers to understand the highest-priority targets for therapist-side adjustments to the WET EBPI when delivered via videoconference-based telehealth. We acknowledge that targeting only therapist-facing challenges is a partial step toward developing a system-wide solution, and ideally, patient and therapist solution development should not be separated in the DDBT framework. Even so, certain VA system constraints necessitated us exploring therapist-identified usability issues before we could address patient-identified issues. As a result, we chose to only present therapist-facing challenges in this section. Given this institutional context, our interview findings yielded the following design constraints for our initial therapist-facing solution:

1. *High reliability for patient care*: in interviews, therapists described resorting to alternatives such as email when the narrative transfer was not readable via other methods even though email was described as less secure, required special permission to execute, and was a temporary fix. Therapists appeared to prioritize delivering WET to veterans even when this practice may not have been in strict compliance with optimal VA security protocols. In addition, therapists described needing to be able to provide support to patients when setting up and using options for narrative transfer.
2. *High flexibility and choice*: in conjunction with the need for high reliability for patient care was the need to offer therapists alternatives when first-line protocols break down. Therapists routinely described having to work through technical and logistical barriers while striving to implement WET protocols as directed. These findings suggest that therapists need fallback alternatives for when these breakdowns inevitably occur.
3. *High reliability for VA patient privacy standards*: in addition to these constraints, discussions with secondary stakeholders and our own experience as VA employees highlighted the need for restricting solutions to VA-approved systems. For instance, an outside tool such as Rocketbook, which digitizes handwritten passages, would not be appropriate as it currently fails to meet VA guidelines for the protection of patient privacy. Thus, our solution would need to be workflow (rather than new tool) focused.

### Discover Phase: Ongoing Work

We are currently recruiting and interviewing veteran patients about their experiences with WET delivered via videoconference-based telehealth. We are in the process of identifying specific facilitators of and barriers to patients’ narrative transfer experiences with their WET telemental health therapists.

### Design and Build Phase: Using Findings to Identify Design Constraints and Develop a Solution

#### Overview

Within the DDBT framework, the *Design and Build* phase begins with information gathered from the *Discover* phase to inform the development of intervention or implementation strategy solutions [1]. Breaking this phase down further, in the *Design* subphase, findings and insights from the *Discover* phase are used to define the initial requirements for potential solutions, and these solutions are then conceived (ie, ideated). These concepts inform the development of low-fidelity (ie, easily modifiable) prototypes in the *Build* subphase, which are then tested with users for feedback and validation. The solutions are refined, which feeds back into another cycle of *Design and Build*. This process is repeated with a subgroup of users until a sufficiently workable solution is derived that can later be tested at scale (and with a broader group of users) in the *Test* phase.

In our interviews, therapists discussed a variety of workarounds for obtaining timely, readable narratives from patients, all of which had barriers to their successful use. For our first attempts at solution design, we decided to focus on narrative transfer as it (1) was the only barrier identified as WET specific, (2) was a critical component in the delivery of WET, and (3) was the biggest barrier to successful web-based delivery identified by therapists. After identifying our constraints, the following design question guided our work for the *Design and Build* phase:

How might we improve usability, security, and readability when transferring handwritten narratives from patients to therapists?

We identified 7 therapist users to work with our UCD expert (author KT) to design and build prototype solutions for narrative transfers. All interactions with these users were individual (ie, no group sessions) and on the web, and only handwritten notes were taken.

#### Data Sources and Methods

Primary users for *Design and Build* work were CRH therapists from group 2 (ie, CRH group) who expressed interest in (and subsequently completed) WET training (*CRH-WET* group). We selected this group to assist in therapist-facing solution development given their combined experience with telehealth care and WET. They worked with our study’s principal investigator (author JCF) and coinvestigator (author SR) to attend WET training conducted by the developers of WET. Training was deemed complete when each CRH-WET therapist identified 2 patient cases and worked through them as training cases with weekly supervision from WET developers. As a result, the therapists’ training completion was staggered. Given this staggered completion, we opted for individual cognitive walkthroughs [24] as a method for iterative prototype development with users. Cognitive walkthroughs are commonly used to identify potential barriers to a particular solution, especially regarding their use in the intended context. This approach is meant to simulate the cognitive processes of users by asking them to think through, out loud, how they might use the solution, thus revealing existing mental models and expectations for use [24].

#### Solution Development

Solution development began with brainstorming ideas (ie, ideation) for narrative transfer, which was conducted with members of the evaluation team and informed by findings from interviews, training observations, and conversations with WET developers and Virtual Care QUERI technical expert panel members. In line with design constraints as outlined previously, during brainstorming sessions, team members often raised concerns about ensuring that solutions adhered to VA regulations for patient privacy and data security, which affected the ideas put forward for development. Multiple potential solutions in the form of therapist-facing workflows were identified during *Design* subphase ideation sessions. These were then explored in *Build* subphase usability testing with CRH-WET users via individual cognitive walkthroughs [24]. Cognitive walkthroughs allowed our UCD expert to meet with the first 2 CRH-WET users to talk through the steps of hypothetically going through each of the solution workflows. Our goal was to obtain feedback on preferences and what users thought of our potential workflows and, ultimately, reduce the number of options presented to subsequent CRH-WET users. The 3 different workflows that were presented to the remaining 5 CRH-WET users were secure messaging via My HealtheVet, My VA Images, and screenshots. Secure messaging via My HealtheVet and screenshots were already in use, as described previously; My VA Images was the only workflow not already in use with our therapist groups and involves the use of mobile app technology that allows patients to share photos or video clips with providers upon the providers’ request. These 5 users also went through cognitive walkthroughs, with each subsequent user’s feedback being integrated to iteratively refine the workflows.

Users recommended that visuals accompany the workflows to facilitate their use in context. Thus, we developed PDF manuals for patients and therapists describing the workflows, which included steps for setting up relevant accounts, ensuring that technological infrastructure was in place, and what to do in the event that a particular workflow could not be executed. The manuals used a combination of graphics and narratives to describe the steps and provide support for executing the workflows. In the case of patient-facing workflows and accompanying manuals, these were intended for therapists to share with patients and will be revised as needed when patient-identified challenges are explored in subsequent evaluation work.

### Test Phase: Ongoing and Planned Work

In the *Test* phase, solutions developed in the *Design and Build* phase are tested at a greater scale, in their intended context and form, and with a greater number of users. In this phase, high-fidelity prototypes are developed and implemented to determine the solution’s feasibility in a “real world” context [1].

Although this paper covers only the work done in the *Discover* and *Design and Build* phases, we want to highlight that the *Test* phase is currently underway in the context of our larger implementation project. We are currently recruiting VA telemental health therapists for a randomized controlled trial designed to compare 2 WET implementation strategies, one of which integrates the workflows and associated manuals developed in this study. The first implementation strategy involves training mental health therapists to deliver WET and includes weekly supervision while the therapist administers the therapy to 2 patients (ie, 2 cases). The second implementation strategy involves the same training or supervision described previously as well as group community of practice calls once each month for 6 months. During the monthly calls, we will share the workflows or manuals with the therapists and troubleshoot narrative sharing and other barriers to WET delivery. Although the primary outcome of the randomized controlled trial is to compare the proportion of patients receiving WET from therapists in the 2 groups, a secondary outcome is the acceptability of the method used to transfer narratives (which should be directly related to the workflows developed in this study).

## Discussion

### Principal Findings

In this formative study, we used qualitative and UCD methods in the first stages of the DDBT framework [1] to identify therapist-facing usability issues and develop a solution for delivering a trauma-focused EBPI, known as WET [9-11], in a VA videoconference-based telehealth context. We found that both therapist user groups—those who deliver WET and those who provide other PTSD therapies via video—experienced challenges with remote workflows, especially when sending narrative transfers or other time-sensitive materials to and from patients. Despite these challenges, therapists perceived WET to be a viable therapy option for patients with PTSD, especially those who were willing to be proactive about addressing their trauma. WET delivered via videoconference-based software was seen as particularly useful given the challenges with COVID-19 in-person appointments (eg, masks preventing reading facial expressions) and the opportunity for patients to write in the perceived comfort of their homes. We used information from therapist interviews to guide preliminary design work related to improving WET narrative transfers, namely, developing workflows that can be used in wider testing with therapists and as a starting point for patient-facing work. In a post–COVID-19 pandemic world in which telehealth becomes ever more ubiquitous, this work has important implications for telehealth therapists within and outside the VA as well as researchers exploring the benefit of integrating user-centered or related design practices with implementation science to improve the usability of EBPIs.

Across both groups of therapists, we found that the challenges described for delivering WET or other PTSD therapies via video appeared in line with previous findings. Our data support prior work showing that WET delivered via videoconference-based telehealth presents challenges such as PTSD therapy avoidance, unreadable handwritten narratives, or difficulty using digital tools (eg, My HealtheVet) [8,12,13]. Our data also support previous findings that narrative account transfers present substantial clinical workflow problems to therapists delivering WET in a telehealth context, and as is common with EBPI adaptations [2], our therapist groups adapted WET and other PTSD therapy processes to their needs, the perceived needs of their patients, and the clinical and institutional context.

Finding ways to adhere closely to the established therapy protocol during video telehealth appointments while ensuring a user-friendly experience for our group of therapists was paramount in our approach. However, user-friendly technological solutions are commonly in conflict with established privacy and security protocols in health care [25,26], and these protocols are frequently perceived as a barrier to the wider adoption of telehealth technologies. Although telehealth and related technologies were in use before the COVID-19 pandemic, the unprecedented rise in remote care services and the temporary loosening of VA regulations have revealed the utility of user-friendly tools in reducing barriers to care delivery [27]. As we heard from our groups of therapists, sometimes the easiest way to execute tasks (ie, emailing handwritten narratives) conflicted with established pre–COVID-19 VA security policies regarding patient privacy but allowed therapists to offer patients timely, needed care. In addition, without appropriate support for their setup and use, formally approved tools such as My HealtheVet, which comply with data security and privacy protocols to protect patients’ information, can be difficult to use for both therapists and patients. Other approaches such as having a technician available to scan and securely send paperwork when patients are seen at an outpatient clinic are viable options for overcoming technological barriers but could not be used for in-home telehealth encounters.

### Ongoing and Future Project Work

We found the DDBT framework to be an efficient, methodical, and user-friendly way to understand certain challenges in delivering WET via videoconference-based telehealth and develop solutions to a specific therapist-facing usability issue, all while remaining in compliance with approved VA rules for protecting patient information. As this evaluation is formative and part of the larger Virtual Care QUERI project, work is ongoing to understand patient-facing challenges and continue to refine therapist workflows based on feedback from additional therapist users. As described in this paper, patient interviews are underway. Therapist-facing solutions are being tested (the *T* in DDBT), and the workflows and accompanying manuals will be continuously revised based on therapist feedback and updated to reflect changes in the underlying software platforms.

### Limitations

Our work has limitations to address. First, as a quality improvement evaluation designed to enhance VA care, the findings have limited generalizability to other contexts or to patients. In addition, although we have designed potential patient workflows, it is important to note that, in the context of this study, these were not developed or tested with patients. Future work needs to include understanding additional barriers to and facilitators of WET video telehealth delivery from the patient perspective and refining and testing patient workflows. However, the findings and design recommendations may be transferable to other contexts, especially when using UCD principles to test their applicability, which prioritize users in unique, local contexts.

Second, all our data collection was remote. This is increasingly common in health services research studies using qualitative methods [28], although UCD typically involves some components of in-person observation and interaction even for interventions delivered via video. Given the continued prevalence and ubiquity of remote work practices [27,28], future work might explore digital ethnographic or other digital qualitative methods for data collection.

Third, we only completed part of the DDBT framework with a portion of users; thus, its applicability across our implementation project is unknown. Future work will be essential to ascertain its full utility, including (1) testing workflows with therapists and patients and exploring other areas for improving WET usability; (2) developing, testing, and scaling an implementation plan for WET video telehealth throughout the VA’s national telemental health program (ie, in CRHs nationwide); and (3) soliciting implementation feedback.

### Conclusions

In a post–COVID-19 world, the need for high-quality and easy-to-implement trauma-focused EBPIs is greater than ever. To provide patients who require mental health interventions with effective, evidence-based care, we must focus on all aspects of their care experience, including the experiences of those people providing them care. Using UCD methods to improve the usability of EBPIs delivered via video is a viable approach to systematically increasing the user-friendliness of such interventions. Exploring the applicability of the DDBT framework to scale tele-EBPIs such as WET is essential for understanding the usefulness of a fully executed UCD approach to improving usability in health care contexts.

## Appendix

Multimedia Appendix 1 Written exposure therapist interview guide.

Multimedia Appendix 2 Clinical Resource Hub therapist interview guide.

## Abbreviations

CPT: cognitive processing therapy
CRH: Clinical Resource Hub
DDBT: Discover, Design and Build, and Test
EBPI: evidence-based psychosocial intervention
PE: prolonged exposure
PTSD: posttraumatic stress disorder
QUERI: Quality Enhancement Research Initiative
UCD: user-centered design
VA: Department of Veterans Affairs
WET: written exposure therapy

## Data Availability

The data sets generated and analyzed during this study are not publicly available because of Veterans Health Administration regulations.
