# Supplementary material for: Improving the Usability of Written Exposure Therapy for Therapists in the Department of Veterans Affairs Telemental Health: Formative Study Using Qualitative and User-Centered Design Methods
Source: JMIR Form Res. 2023 Nov 6;7:e47189. doi: 10.2196/47189 (PMC10660215; doi:10.2196/47189)
Supplement: Multimedia Appendix 1 [file formative_v7i1e47189_app1.docx]

**Appendix A:** Interview questions and follow up probes for written exposure therapy (WET) providers.

**INTERVIEW QUESTIONS** *(note: this is a semi-structured protocol. Exact wording and order of questions may be adapted during the interview, and interviewer may ask follow-up questions as needed.)*

***Background***

**How long have you been providing WET?**

- Have you done it in person or only virtually?
- Overall, what are your impressions of the therapy? Have you found it useful?
- How long have you been treating PTSD generally (apart from WET)

***Learn about their process and experience***

**Walk me through your process for conducting WET virtually.** (Alternate wording: Tell me about the process for the last patient you treated.)

- How do you decide when to use WET for a patient?
- Where does the process start?
  - *(Ask about measures like PCL, SUDS, other metrics taken before, during, or after the session)*
- What happens next?
  - *(Include questions about how they give instructions to patient.)*
- What do you do while the patient is writing?
- How do you receive the written passage/narrative from the patient?
- Is there any difficulty getting the patient to send you the passage?
- Is there any difficulty accessing or reading the passage?
- What do you do after you read the passage?
- What happens to the passage after a session?
- When do you review the passage and how do you record any feedback you want to provide?
- What kind of feedback on the passage do you provide to the patient? Can you give me an example?
  - Is it ever difficult to decide what kind of feedback to provide to the patient? Tell me more.
  - Are subsequent sessions tailored based on passage feedback?
- Where does the process stop?
- Think about everything you need to do for a patient session. How long does this process take, end to end?
- For example, reading the passage from the last session, deciding what on feedback to provide, reviewing the prompts, completing clinical documentation before and after the session, etc.
- **What happens when all WET sessions are completed?**
- Are any other people at the clinic involved in this process? Who? What is their role?
- **Have you ever had to change or adapt the WET protocol for any reason?**
- Why/how did you adapt the protocol?
- How often do you have to adapt the protocol?
- Have you had a patient drop out? How was this handled? Can you explain the circumstances?
- If you have a patient who is particularly distressed or emotionally activated, how do you respond?

**Where are you working these days (physically)?**

- Can you show me your workspace through the computer camera?

*Alternative line of questions:*

- Tell me how the space you work in relates to providing this therapy?
- Describe your ideal workspace?
- Is the space you are currently in situated for this therapy? Can you tell me more about that?

Why/why not?

- What do you need in terms of your environment to complete a successful WET session with a patient? Is this your current workspace set up?
- **Have you experienced distractions or interruptions during the virtual WET sessions?** (e.g. while the patient is supposed to be writing?)
- What kind?
- What impact does that have on the sessions?
- How do you recover from those interruptions?
- Have you found any strategies to prevent this kind of interruption?

***Additional Information***

**How much does this process vary between patients?**

- Can you give me some examples?

**[If applicable] How does this process compare to when you did this in person?**

- Probe on specific topics: stakeholder involvement, handoffs, distractions, interaction over the written passage, etc.

**What would you change about this process if you could?** (What would your ideal version look like?)

- Imagine a magic button, and if you pressed it, you can make the WET process work exactly as you want it to. What happens when you press the button?
