# Supplementary material for: Improving the Usability of Written Exposure Therapy for Therapists in the Department of Veterans Affairs Telemental Health: Formative Study Using Qualitative and User-Centered Design Methods
Source: JMIR Form Res. 2023 Nov 6;7:e47189. doi: 10.2196/47189 (PMC10660215; doi:10.2196/47189)
Supplement: Multimedia Appendix 2 [file formative_v7i1e47189_app2.docx]

**Appendix B:** Interview questions and follow up probes for clinical resource hub (CRH) providers.

***Background***

**What’s your role?** (Type of provider? Family medicine vs. Internal med vs. psych vs. msw?)

- If applicable: How often would you say you treat patients for mental health issues?
- What kinds of mental health issues do you usually see?

***Learn about their process and experience***

**Walk me through your process for treating someone with {PTSD/some other condition if they don’t treat PTSD, like depression}.** (Alt wording: Tell me about the process for the last patient you treated.)

- Where does the process start?
- What happens next?
- What information do you get about the patient before you see them?
- Do other people in the clinic interact with the patient before you?
- Are any other people at the clinic involved in this process? Who? What is their role?
- At what point do you feel like you need to refer the patient to another clinician?
- Do patients ever have to send you information between appointments? (e.g., measurement-based care?)
  - If so, have you ever had any difficulties getting that information?
    - If so, what happened? How did you overcome those difficulties?

**Describe the end of your process with a patient.**

- What does your last interaction with the patient look like?
- What kinds of wrapping-up tasks do you do after that?
  - What does follow up care look like?
  - If follow up is not with you, what is a common handoff? (e.g. referral to specialist, etc.)

**Where are you working these days (physically)?** (Are you seeing people from home or from a clinic right now?)

- Can you show me your workspace through the computer camera?

*Alternative line of questions:*

- Tell me how the space you work in relates to providing care for your patients?
- Describe your ideal workspace?
- Is the space you are currently in best situated for your work? Can you tell me more about that? Why/why not?
- What do you need in terms of your environment to successful connect with patients? Is this your current workspace set up?
- **Do you ever experience any distractions or interruptions during virtual patient sessions?**
- What kind?
- What impact does that have on the sessions?
- How do you recover from those interruptions?
- Have you found any strategies to prevent this kind of interruption?

**Are you familiar with the WET protocol already?**

- How familiar/what do you know about it?
- If applicable: Do you have any questions/concerns/apprehension about conducting WET virtually?

***Additional Information***

**[If applicable] How does this process compare to when you did this in the clinic?**

- Probe on specific topics: stakeholder involvement, handoffs, distractions, rapport...

**What would you change about this process if you could?** (What would your ideal version look like?)

- Imagine a magic button, and if you pressed it, you can make the virtual treatment process work exactly as you want it to. What happens when you press the button?
- Are there places where you feel like you would want more support or training (in terms of caring for PTSD patients)?
